# Metacognitive Labeling of Contentious Claims: Facts, Opinions, and Conspiracy Theories

**DOI:** 10.3389/fpsyg.2021.644657

**Published:** 2021-03-25

**Authors:** Robert Brotherton, Lisa K. Son

**Affiliations:** Department of Psychology, Barnard College, Columbia University, New York, NY, United States

**Keywords:** conspiracy theories, metacognition, beliefs, politics, misinformation, motivated cognition

## Abstract

Congenial information is often judged to be more valid than uncongenial (but otherwise equivalent) information. The present research explores a related possibility concerning the process by which people label a claim as fundamentally factual (open to proof or disproof) or opinion (a matter of personal preference not amenable to falsification). Rather than merely being more skeptical of uncongenial claims, uncongenial claims may be metacognitively categorized as more opinion than factual, while congenial claims may be more likely to be categorized as factual. The two studies reported here attempt to trace a preliminary outline of how claims are categorized as fact, opinion, or some mix of the two in the context of mundane claims, contentious political issues, and conspiracy theories. The findings suggest that claims are more likely to be labeled factual (and, to a lesser extent, are less likely to be labeled opinion) to the extent that one subjectively agrees with the content of the claim. Conspiracy theories appear to occupy a middle-ground between fact and opinion. This metacognitive approach may help shed light on popular debate about conspiracy theories, as well as seemingly intractable political disagreements more generally, which may reflect fundamental differences in the perceived epistemic foundations of claims rather than simple disagreement over the facts of the matter. Given limitations of the stimuli and participant samples, however, it remains to be seen how generalizable these findings are.

## 1. Introduction

“I had to deny knowledge in order to make room for faith.”

—Immanuel Kant, *Critique of Pure Reason*, 1781

“You are very fake news.”

—T-shirt slogan, 2018

Information and experiences are frequently open to interpretation. Hastorf and Cantril (1954) demonstrated that a rough college football game was interpreted substantively differently by supporters of each side; one side tended to see a fair game while the other saw unsporting behavior from the rival team. Kahan et al. (2012) demonstrated a similar phenomenon experimentally, using video footage of a politically contentious protest and manipulating participants' understanding of the protesters' stance. Participants' perceptions of the protest tended to align with their prior political convictions. Those who disagreed with the stance of the protesters saw their behavior as unacceptably disruptive, and potentially even violent, while those who agreed with the stance of the protesters saw it as a non-violent exercise of freedom of speech. This kind of motivated political reasoning has been demonstrated in many studies (Ditto et al., 2019). Diverging interpretations of information arguably reflects *motivated* cognition, in which prior beliefs influence the perceived meaning or validity of information (e.g Dunning, 2015). Yet *meta*cognition may also play a role. Rather than simply questioning the validity of uncongenial facts, partisans may categorize uncongenial claims as fundamentally less *matters of fact* (which can be proven or disproven) than articles of *opinion* or *faith* (expressions of personal preference or ideology which cannot be proven or disproven). Similarly, congenial opinions or ideological statements may take on the appearance of fact. To date, little research has investigated the metacognitive processes underlying the subjective categorization of statements as fact and opinion. How does one determine whether a claim one encounters—or a belief of one's own—is factual knowledge, personal opinion, or an article of faith? The current research attempts to trace a preliminary outline of this metacognitive belief-categorization process in the context of mundane claims, contentious political issues, and conspiracy theories.

Kant (1781) articulated the traditional wisdom that there are three ways of believing something to be true: possessing factual knowledge (Wissen), holding an opinion (Meinen), and maintaining faith (Glauben) (see Stevenson, 2003). Contemporary psychological research provides evidence that such a distinction is psychologically meaningful and consequential. Children as young as five differentiate statements of fact, opinion, and religious belief, allowing that different people can hold differing opinions but that in disagreements over matters of fact typically only one person can be right (Heiphetz et al., 2013). Children and adults also perceive the different categories of expression as revealing different qualities about the speaker and about the world. For example, “The Nile is the longest river,” is judged to reveal something about the world, but little about the personal characteristics of the claimant. “The Nile is the most beautiful river,” appears less informative about the world, but more revealing about the preferences of the claimant (Heiphetz et al., 2014). Correspondingly, opinions are judged to be more biologically-based while factual beliefs are seen as learned (Heiphetz et al., 2017). Religious claims, such as “God answers prayers,” appear to occupy a middle-ground between fact and opinion, intermediate in terms of their perceived basis in biology, openness to disagreement, and providing information both about the world and about the characteristics of the speaker (Heiphetz et al., 2013, 2014, 2017; see also Van Leeuwen, 2014; Levy, 2017).

Such research supports the distinction between modes of belief articulated by Kant (1781), and raises questions about *how* individuals categorize statements as factual, opinion, or a matter of faith. Metacognition, at its core, is knowledge about one's own knowledge; i.e., a person's ability to evaluate their own thoughts and to organize the information that they receive (Dunlosky and Bjork, 2008; Metcalfe et al., 2020). Our metacognition faculties allow us to discriminate between what we know and do not know (Metcalfe and Son, 2012; Kornell and Finn, 2016), what we can learn and what may be impossible to learn (Son and Sethi, 2010; Bae et al., 2020), what is real and what is imagined (Buda et al., 2011; Dehaene et al., 2017), and in the current examination, what is fact and what is opinion.

Research examining the developmental trajectory of the ability to differentiate factual, opinion-based, or religious statements has used deliberately simple, unambiguous stimulus statements (Heiphetz et al., 2013, 2014, 2017). In everyday life, labeling claims as factual or opinion is more challenging, presenting opportunities for miscategorization. A survey by the Pew Research Center examined Americans' perceptions of realistic political statements (Mitchell et al., 2018). Participants were asked, “Regardless of how knowledgeable you are about the topic, would you consider [each] statement to be a factual statement (whether you think it is accurate or not) OR an opinion statement (whether you agree with it or not)?” Despite the clear distinction, respondents frequently miscategorized factual claims as opinion and vice-versa. For example, 44% labeled the factual statement “Spending on Social Security, Medicare, and Medicaid make up the largest portion of the US federal budget” an opinion. Likewise, 29% labeled the opinion statement “Democracy is the greatest form of government” as factual. Moreover, respondents' partisanship influenced how they labeled claims. Democrats were more likely than Republicans to mislabel “Increasing the federal minimum wage to $15 an hour is essential for the health of the US economy” as a factual claim, while Republicans were more likely to mislabel “Government is almost always wasteful and inefficient” as factual.

(Perceived) political misinformation presents an even more contentious epistemic domain. The term “fake news” has become a popular method of contesting the epistemic status of uncongenial claims. Studies obtaining top-of-mind associations (van der Linden et al., 2020) and using experimental methods (Harper and Baguley, 2019) suggest that partisans across the political spectrum use the term in an ideologically-motivated way, to dispute the factuality (not merely the veracity) of uncongenial information. Likewise, liberals and conservatives tend to selectively question the credibility of scientific findings when they find the conclusions disagreeable (Washburn and Skitka, 2018).

Conspiracy theories—unproven claims about the existence of nefarious secret plots (see Brotherton, 2013)—present another divisive epistemic domain. Endorsement of conspiracy theories is widespread (Oliver and Wood, 2014) and a product, in part, of ubiquitous and adaptive psychological phenomena, such as the attribution of agency to ambiguous events (e.g., Brotherton and French, 2015; Douglas et al., 2016), yet conspiracy theorizing is popularly portrayed as misguided at best, if not outright ridiculous and dangerous at worst (e.g., Boot, 2020). Given that claims of conspiracy inherently concern ostensibly hidden information, conspiracy theories necessarily blend factual claims about known events with speculation about concealed actions and the alleged conspirators' motives. Moreover, adherents are in at least some cases somewhat open to mutually-contradictory narratives (Wood et al., 2012), and endorsement of fictitious and historically accurate allegations of conspiracy are strongly correlated (Wood, 2016), suggesting that acceptance or rejection of conspiracy theories may depend on individuals' broader conspiracist *ideology*. This blending of factual claims, ideological conviction, and opinionated speculation may position conspiracy theories somewhere between pure fact and pure opinion.

Conspiracy theories and political misinformation more broadly may hold a stronger appeal during times of crisis (van Prooijen and Douglas, 2017), including the Covid-19 pandemic. Contemporaneous research suggests that support for public health recommendations and vaccine-uptake intentions, for example, are predicted by attitudes toward related misinformation and conspiracy theories, as well as by generic conspiracist ideation (Enders et al., 2020; Fazio et al., 2020; Miller, 2020; Romer and Jamieson, 2020; Uscinski et al., 2020). The true prevalence (Freeman et al., 2020a,b; Sutton and Douglas, 2020) and behavioral effects (Earnshaw et al., 2020) of such beliefs are difficult to establish. Yet given the potential influence of conspiracy theories and misinformation on the course of a public health crisis, and on trust and participation in the political process more generally (Invernizzi and Mohamed, 2019), it is important to understand not just who endorses and who rejects such claims, but how people metacognitively categorize the claims. Are conspiracy theories and misinformation seen as more fact-like, more opinion-like, or a mixture of the two, as other ideological claims appear to be (Heiphetz et al., 2013, 2014, 2017)? The distinction may have implications for strategies to address mistaken beliefs; fact-checking has been shown to be a somewhat successful strategy for correcting specific mistaken factual beliefs (Wood and Porter, 2019), though less impactful in changing people's broader ideological positions (Nyhan et al., 2019).

In sum, the challenge of labeling one's own beliefs, or someone else's statements, as fact or opinion is a pervasive, under-acknowledged, and potentially consequential aspect of metacognition. Important questions remain about how this metacognitive process operates. A question of particular interest, given the existing literature on motivated reasoning, is the extent to which labeling claims as factual depends not solely on their epistemological amenability to (dis)confirmation, but on the degree to which one agrees or disagrees with the statement. The exploratory studies reported here are intended as a preliminary examination of these questions.

## 2. Study 1

This initial study was intended as a first step toward examining the perception of statements as variously factual or opinion-based, and how this corresponds to subjective agreement and knowledge of the claims, outside of the contentious context of politics. Whereas, previous research has used unambiguously factual or opinion-based statements as stimuli, this study used intentionally broad statements which could feasibly be seen as fact and opinion (e.g., “Hard work pays off”). We have participants rate each statement according to how factual or opinion-based it is, as well as obtaining ratings of the participants' own agreement and knowledge of the claim.

This allows us to descriptively address several preliminary questions. First, are the labels *fact* and *opinion* categorical distinctions or a matter of degree? That is, are *fact* and *opinion* ratings on a numerical scale normally distributed, or, on the contrary, bimodal distributions clustered at each end of the rating scale. Relatedly, are the labels mutually exclusive? That is, can a statement be perceived as both somewhat factual and somewhat opinion-based? Lastly, to what extent are fact/opinion ratings influenced by participants' agreement with each statement, and their self-rated knowledge of the general topic?

### 2.1. Method

#### 2.1.1. Participants

Participants were recruited via Barnard College's Introduction to Psychology undergraduate research participation pool.^1^ A total of 211 participants provided complete data (16 participants did not complete the procedure). As participants were exclusively undergraduate students, 88% were aged between 18 and 21 (age information was missing for three participants). As Barnard College is a women's college, 86% of participants were female (gender information was not provided by one participant; male participants are accounted for by Columbia University students who can enroll in Barnard courses). Most participants (74%) indicated USA as their nationality.

#### 2.1.2. Materials

**Figure 1:**
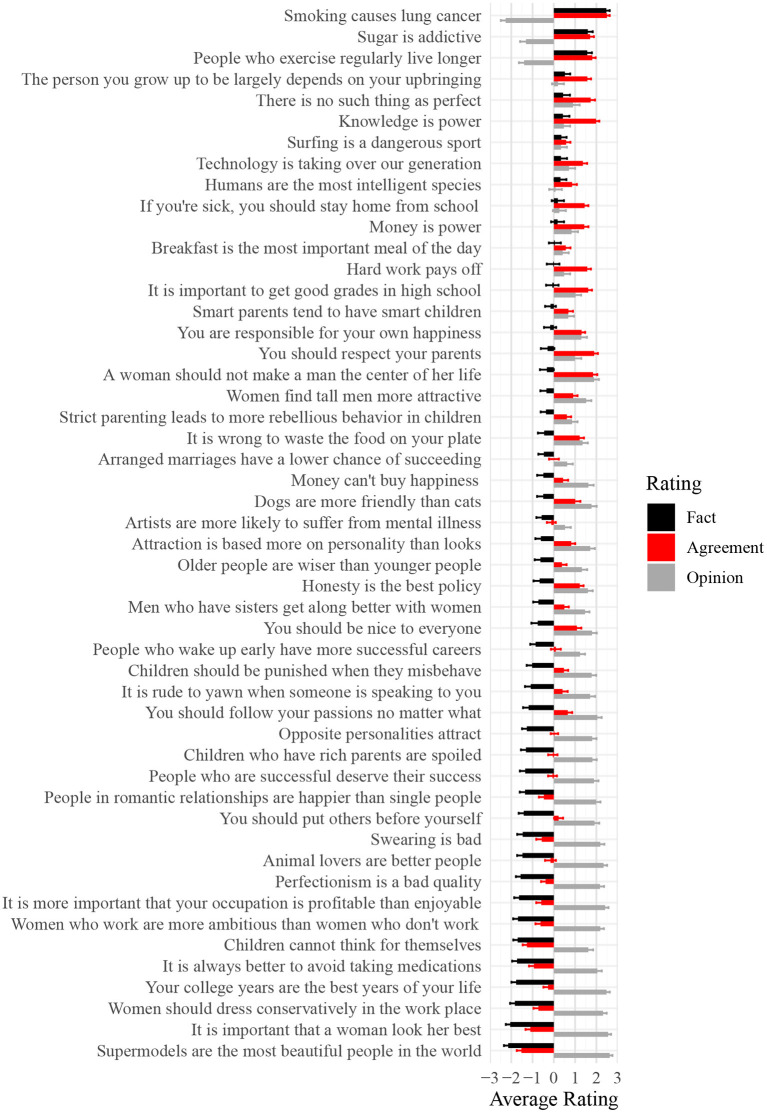
Average fact, opinion, and agreement ratings for each of the fifty statements used in Study 1. Scores of −3 and +3, respectively correspond to the labels “This is not a fact” and “This is a fact” for Fact ratings; “This is not an opinion” and “This is an opinion” for Opinion ratings; and “I completely disagree” and “I completed agree” for Agreement ratings.

Fifty statements were written to serve as stimuli. The statements were intended to reflect a wide range of claims and beliefs, e.g., “Smoking causes lung cancer”; “Money is power”; “Swearing is bad” (see Figure 1 for full wording of all statements).

Every participant rated each statement four times, each time using a different 7-point rating scale. For *factual* ratings, the scale endpoints were labeled “This is not a fact,” and “This is a fact.” For *opinion* ratings, the scale endpoints were labeled “This is not an opinion,” and “This is an opinion.” For *agreement* ratings, the scale endpoints were labeled “I completely disagree,” and “I completely agree.” For *knowledge* ratings, the scale endpoints were labeled “I know very little about this topic,” and “I know a lot about this topic.”

Demographic questions asked participants to indicate their age (in years), nationality (USA, Korea, or other), and gender (female, male, or non-binary/other).

#### 2.1.3. Procedure

After providing informed consent and answering the demographic questions, participants were asked to read the following instruction:

In this study, we are interested in how people respond to the things other people say. You will read about some things that someone else might say. After you read about what each person says, please use the scales provided to indicate your response.

Participants were then presented with stimuli statements, one at a time, via a computer-based Qualtrics survey. Each statement was prefaced with “Someone says that,” i.e., “Someone says that women find tall men more attractive.” Each statement was repeated a total of four times, once for each of the four rating scales. Statements were presented in blocks according to the rating scales. Each block began with an instruction in the form “In this section of the study, we would like to know how much you think what each person says is a FACT. Please use the scales provided to indicate how much you think what each person says is a fact.” The repeated presentation of statements with separate rating scales (as opposed to simply bundling the four ratings in a single presentation of each statement) was intended to disentangle ratings, avoiding any potential implication that the rating scales should be treated as mutually exclusive.

The *factual, opinion*, and *agreement* blocks were presented in random order. The *knowledge* block was always presented last. This was intended to mitigate a potential order effect whereby having participants reflect on their knowledge of a claim could influence their *factual, opinion*, or *agreement* ratings. Within blocks, statement order was random.

After completing the procedure, participants were thanked and debriefed.

#### 2.1.4. Data Analysis

We used R (Version 4.0.0; R Core Team, 2019) for all our analyses.

### 2.2. Results and Discussion

Responses to the 7-point *factual, opinion, knowledge*, and *agreement* rating scales were re-coded by subtracting four points from each, so that scores were centered on zero and ranged from −3 to +3.

**Figure 2:**
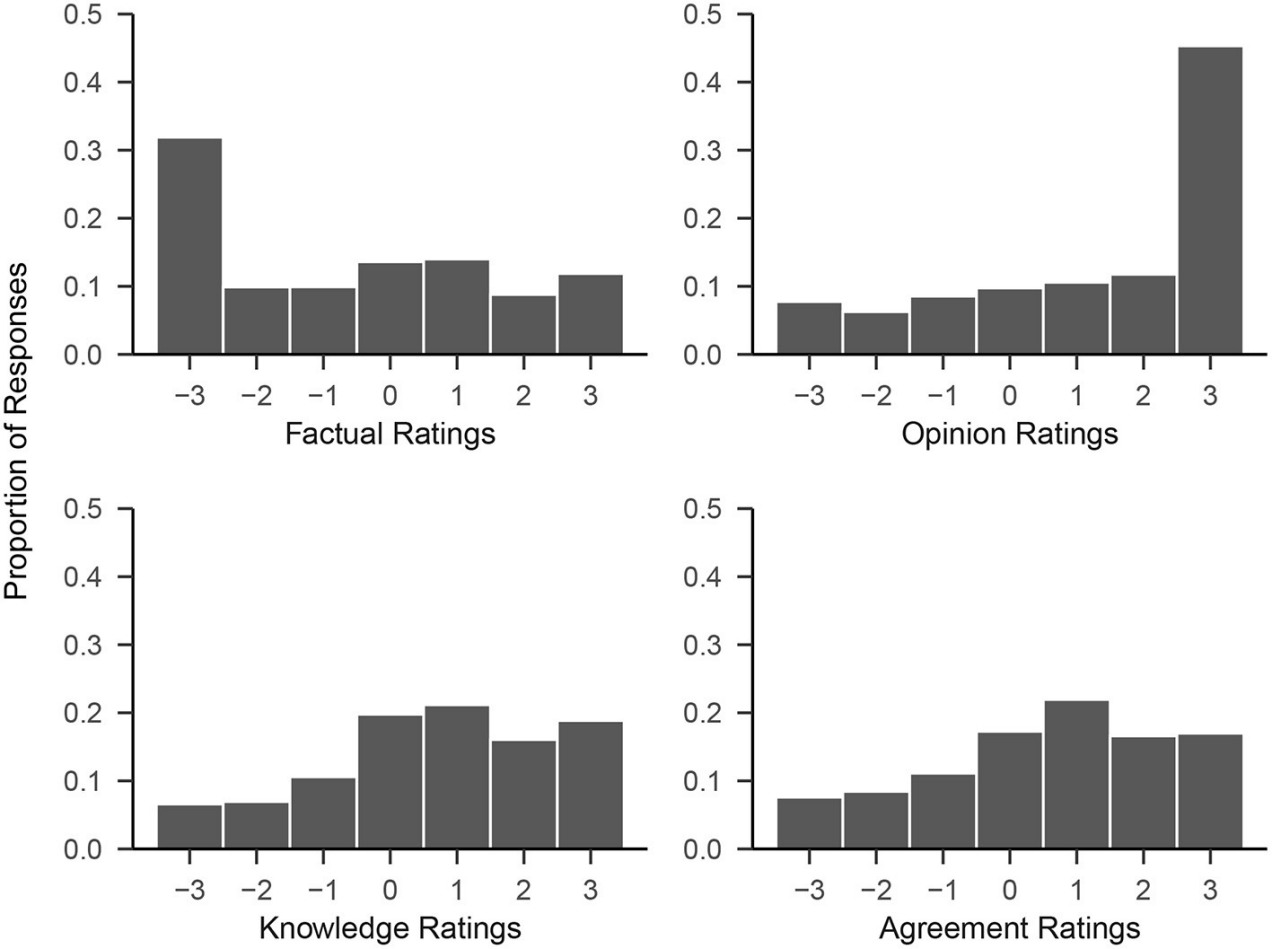
Histograms of responses to all 50 statements across Fact, Opinion, Knowledge, and Agreement rating scales in Study 1. Scores of −3 correspond to the labels “This is not a fact,” “This is not an opinion,” “I know very little about this topic,” and “I completely disagree,” respectively. Scores of +3 correspond to the labels “This is a fact,” “This is an opinion,” and “I know a lot about this topic,” and “I completely agree.” Intermediate scale points were not verbally labeled.

We first produced histograms of every participant's ratings for each of the four rating blocks to visualize the distributions of ratings (see Figure 2). As each participant provided 50 ratings per rating scale, the total number of data points for each histogram is N*50 = 10, 550. Self-rated knowledge and agreement were approximately normally distributed, with slight negative skew, and centered around a rating of 1, just above the mid-point of the scale. *Factual* ratings were strongly positively skewed; ~32% of ratings were the lowest possible scale value (which had the verbal designation “This is not a fact”). *Opinion* ratings were strongly skewed in the opposite direction; 45% of all ratings were the highest possible response option (labeled “This is an opinion.”). This suggests that the labels *fact* and *opinion* were to a substantial (but not complete) extent used categorically; participants often rated statements as entirely opinion and entirely non-factual. That said, participants did display some openness to the idea of varying degrees of opinion- and fact-ness. The various intermediate options combined accounted for more than half of all *factual* and *opinion* ratings, respectively.

As an initial summary of the associations among the different ratings we obtained for each statement, we calculated each statement's average rating within each of the four rating blocks. Figure 1 shows averages for the *factual, opinion*, and *agreement* blocks (*knowledge* ratings omitted for visual clarity). Higher *opinion* ratings appear to generally correspond to lower *factual* ratings, suggesting that on the whole participants used the labels as mutually-exclusive categories (these visual trends are supported by the correlation coefficients presented in Table 1). The graph also suggests a correspondence between the extent that a statement is perceived to be factual, on average, and the degree to which participants subjectively agree with it. At the extremes, “Smoking causes lung cancer” was rated as strongly factual (*M* = 2.47) and garnered equally strong average agreement (*M* = 2.50); “Supermodels are the most beautiful people in the world” was rated as strongly not factual (*M* = −2.15), and garnered strong disagreement (*M* = −1.53). However, there were some statements for which participants leaned toward agreement while seeing the statement as not factual, on balance (e.g., “Dogs are more friendly than cats”: *M*_*factual*_ = −0.50; *M*_*agreement*_ = 1.00). *Opinion* ratings appear to be more extreme than *factual* ratings overall. Approximately half of the 50 statements garnered average *opinion* ratings approaching or exceeding 2 (out of a maximum re-coded score of 3). Only three of the 50 statements garnered similarly extreme *factual* ratings.

**Table 1:** Pearson's correlation coefficients for Studies 1 and 2.

	**Study 1**	**Study 2**		
		**Type of statement**		
**Pairwise comparison of ratings**		**Fact**	**Opinion**	**Conspiracy**
Factual × Opinion	−0.52	−0.76	−0.82	−0.81
Factual × Knowledge	0.22	0.08	*0.04*	*0.02*
Factual × Agreement	0.52	0.35	0.31	0.27
Opinion × Knowledge	−0.13	*−0.02*	*−0.01*	0.06
Opinion × Agreement	−0.38	−0.24	−0.17	−0.12
Knowledge × Agreement	0.22	0.09	0.10	*0.04*

*Given the exploratory nature of the analyses, Pearson's correlation values are given as a descriptive statistic here. For completeness, however, we note that, given the large number of rating pairs per pairwise comparison (10,550 for Study 1; 1,451–1,458 for Study 2), all correlations for Study 1 are statistically significant at the level p < 0.05. For Study 2, correlations greater than r≈0.05 are significant at the level p < 0.05. Non-significant correlations (p>0.05) are shown in italics*.

**Figure 3:**
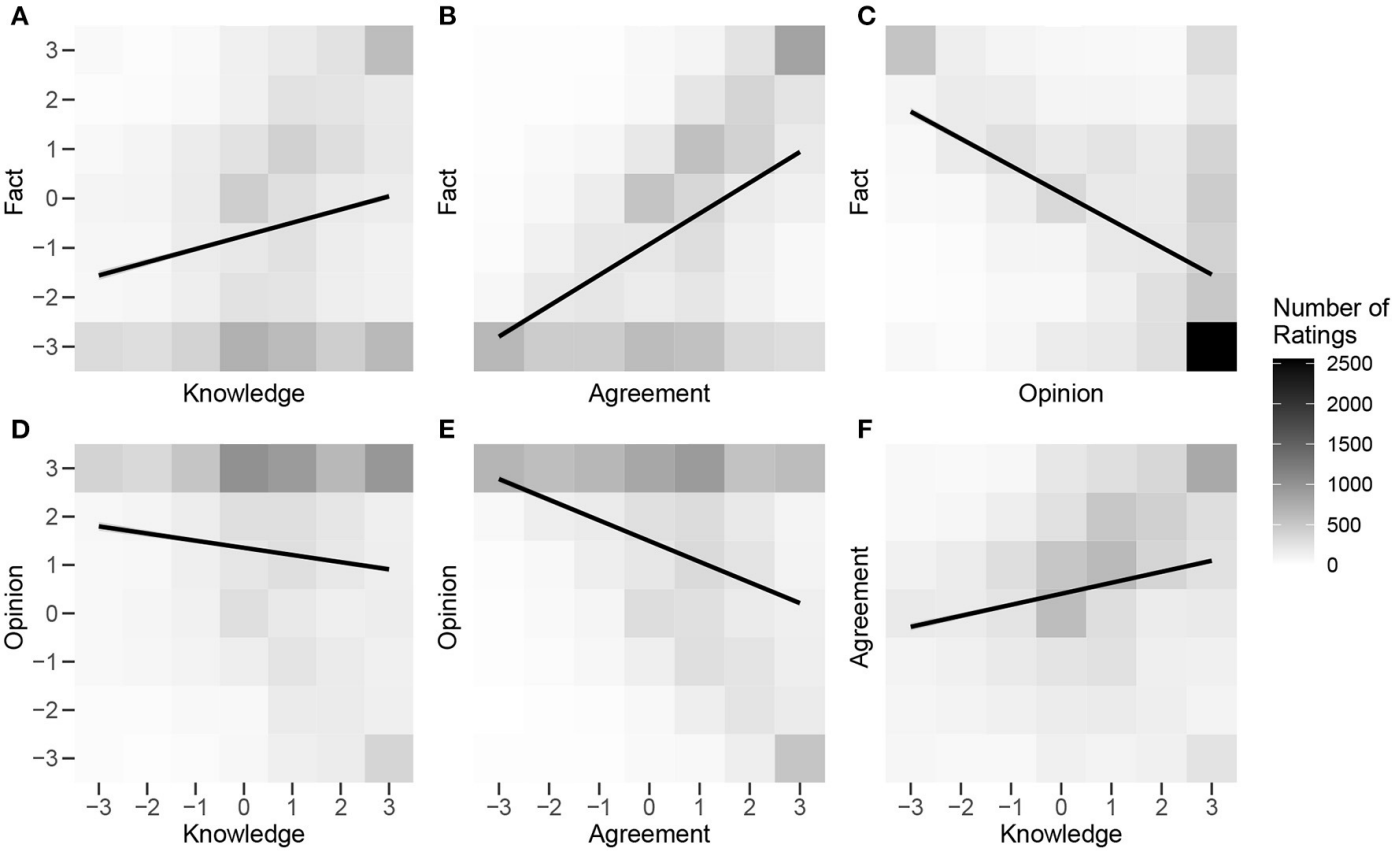
Two-dimensional histograms of pairwise associations between Fact × Knowledge **(A)**, Fact × Agreement **(B)**, Fact × Opinion **(C)**, Opinion × Knowledge **(D)**, Opinion × Agreement **(E)**, and Agreement × Knowledge **(F)** rating scales for Study 1. The shading of each cell represents the number of paired ratings falling into that category, with darker cells representing more ratings. Scores of −3 and +3 correspond to the labels “This is not a fact” and “This is a fact” for the Fact rating scale; “This is not an opinion” and “This is an opinion” for the Opinion rating scale; “I know very little about this topic” and “I know a lot about this topic” for the Knowledge rating scale; and “I completely disagree” and “I completely agree” for the Agreement rating scale. Intermediate scale points were not verbally labeled.

Next, bivariate correlations were computed to establish the strength of relationships between pairs of ratings. Pearson's *r* correlation coefficients were calculated for all six possible pairwise combinations of the four rating scales, using every pair of 10,550 ratings from all 211 participants (see Table 1). To visualize these pairwise associations and further clarify the trends and skews within the data, we generated a 2-dimensional histogram for each of the six pairwise comparisons (see Figure 3). The shading of each square represents a count of the number of observations within a particular area of the 2-dimensional space. Linear fit lines are also shown. There was a moderate negative correlation between *fact* and *opinion* ratings (Figure 3C), confirming that the labels are to some extent used in a mutually exclusive way. That is, the more a claim is seen as factual, the less it is seen as an opinion, and vice versa. Yet this appears to be due, to a large extent, to the large number of instances in which a participant rated a statement as completely opinion and not at all factual (close to 25% of all rating pairs). In comparison, participants rated a statement as both maximally opinion *and* factual < 3% of the time. Focusing on the role of subjective agreement in labeling a claim as factual or opinion (Figures 3B,E), the correlations suggest that agreeing with a claim predicts labeling it more factual and less of an opinion. This association was stronger for *factual* ratings, however; participants were more willing to call something they agreed with an opinion than to call something they disagreed with factual, suggesting potential asymmetry in how agreement predicts perceptions of the extent to which a statement is factual or opinion. Participants' self-rated knowledge also played a role in labeling claims as factual or opinion (Figures 3A,D), with greater knowledge correlating positively with factual ratings and negatively with opinion ratings, though to a lesser extent than subjective agreement. There was also a weak positive correlation between self-rated knowledge and agreement with the statements (Figure 3F).

In sum, the main findings of note to emerge from Study 1 were, first, that people appear to use “fact” and “opinion” as somewhat categorical and mutually-exclusive labels. Second, there was a moderate-to-strong correlation between agreement and fact ratings, and a negative (albeit weaker) correlation between agreement and opinion ratings. That is, people are more inclined to call a statement factual the more they agree that it is true. Likewise, people are more inclined to call a statement opinion when they disagree with it.

However, it is unclear whether the skew toward labeling statements as opinion and not factual represents a general preference for calling ambiguous statements opinions, or the statements used simply leaned toward opinion rather than fact. Importantly, the wording of the *factual* rating scale may have also influenced judgments by failing to make clear the intended definition of *factual* (as in *a statement amenable to proof or disproof* ) as distinct from colloquial use which equates *fact* and *true statement*. The correlation between subjective agreement and perceived factuality may reflect this interpretation of the word *fact* rather than metacognitive mislabeling driven by subjective (dis)agreement.

## 3. Study 2

This second study was intended to examine whether the relationships observed in Study 1 hold for a different set of stimuli statements, and with clarified instructions intended to disentangle being *factual* from being *true*. Whereas, Study 1 employed relatively neutral statements, this study employed overtly political statements. Additionally, whereas the statements used in Study 1 were intended to be of somewhat ambiguous epistemic footing, this second study followed previous research in using statements which were definitively factual or opinion-based, according to criteria used in previous research (factual statements are able to be proven or disproven using objective evidence, whereas opinion statements are not; Mitchell et al., 2018).

To improve upon Study 1, efforts were made in both the general instructions to participants and the wording of the rating scales to make this working definition clear, emphasizing that a statement one perceives to be *wrong* could still be classified as *factual*.

Moreover, the current study included a third category of statement: conspiracy theories (statements which refer to the secret, nefarious actions of a group of people; see Brotherton, 2013). This addition was intended to shed light on whether conspiracy theories are generally seen as claims of fact, of opinion, or as a distinct category occupying the space between fact and opinion, akin to religious claims in previous work (Heiphetz et al., 2013, 2014, 2017).

### 3.1. Method

#### 3.1.1. Participants

Participants were recruited via Barnard College's Introduction to Psychology undergraduate research participation pool students participated in return for course credit. A total of 146 participants provided complete data (Of 183 people who began the survey, five did not complete the procedure; 27 failed an attention check and were excluded).

As participants were exclusively undergraduate students, 95% were aged between 18 and 21. As Barnard College is a women's college, 92% of participants were female (male participants are accounted for by Columbia University students who can enroll in Barnard courses.) Most participants (88%) indicated USA as their nationality.

Politically, the sample leaned toward liberal ideology; 40% said they identified as a “Strong liberal,” 38% as a “Moderate liberal,” 18% as “Independent,” 4% said they identified as a “Moderate conservative,” and 0% identified as a “Strong conservative.”

#### 3.1.2. Materials

A total of 60 statements were generated to serve as stimuli for this study (see Supplemental Material for full wording of all statements). These spanned 10 topics (e.g., immigration, gun control, climate change). For each topic, two statements of fact, two statements of opinion, and two statements of conspiracy were written. For example, “Global temperatures have risen more than 2 degrees Fahrenheit since 1900” (statement of fact); “Climate change is an existential threat” (statement of opinion); “The scientific consensus about climate change is distorted by scientists' own interests” (statement of conspiracy). The two versions of each type of statement were intended to be approximate negations of one another: “Global temperatures have risen less than 2 degrees Fahrenheit since 1900” (fact); “Climate change is not an existential threat” (opinion); “The scientific consensus about climate change is not distorted by scientists' own interests” (conspiracy). Which version of a statement a participant saw was manipulated between-participants; i.e., each participant saw one or the other version. Given the expected ideological homogeneity of the participant sample, this was intended to maximize variability in the data by ensuring a range of agreement and disagreement across the two versions of each statement.

Participants rated each statement on four rating scales, presented in the following order: *Knowledge* (How KNOWLEDGEABLE are you about this topic?); *Agreement* (Regardless of how knowledgeable you are about the topic… How much do you AGREE with the statement/think it is ACCURATE?); *Factual* (Regardless of whether you agree/think it is accurate or not… Would you consider this statement to be a FACTUAL statement?); and *Opinion* (Regardless of whether you agree/think it is accurate or not… Would you consider this statement to be an OPINION statement?). Each scale was rated on a scale from 1 (Not at all) to 5 (Completely).

Additional questions asked participants to indicate their age (in years), nationality, gender, and political ideology (phrased as “Do you consider yourself politically…” followed by the options Strong liberal, Moderate liberal, Independent, Moderate conservative, Strong conservative).

#### 3.1.3. Procedure

To clarify the meaning of the “factual” and “opinion” rating scales for the purposes of the study, participants were asked to read the following instructions, modeled on those of Mitchell et al. (2018):

Generally, a statement would be considered a FACTUAL statement to the extent that you think that the statement could be proved or disproved based on objective evidence, regardless of whether you think the statement is accurate or not.

A statement would be considered an OPINION statement to the extent that you think that it was based on the values and beliefs of the person making the statement and could not definitively be proved or disproved based on objective evidence, regardless of whether you agree with the statement or not.

So, for example… “The Hudson River is the world's longest river.”

…would be more of a FACTUAL statement, even though it is not true, because it could be proved or disproved.

“The Hudson River is the world's most beautiful river.”

…would be more of an OPINION statement, since it is based on someone's values or beliefs and could not be definitively proved or disproved.

In this study you will see a series of statements that you might hear someone say.

For each statement, we will ask you to rate it according to the four questions below, asking how KNOWLEDGEABLE you are about the topic; how much you AGREE with the statement/think it is ACCURATE; how much you consider it a FACTUAL statement; and how much you consider it an OPINION statement.

There are no right or wrong answers - we are interested only in your intuitive response to each statement.

This was followed immediately by an attention check question. The four rating scales were presented, but the instructions read “If you have read and understood these instructions, please select Completely (5) for each of the four questions here, and then click next to move on with the study.” As noted, 27 participants failed the attention check (16% of the sample).

Participants were then shown the stimuli statements, one at a time, via a computer-based Qualtrics survey. While Study 1 prefaced statements with “Someone says that…,” for this study statements were merely enclosed within quotation marks, i.e., “The pharmaceutical industry has the largest lobby in congress.” The four rating scales were presented together, on the same page as the statement itself. The statements were presented in random order. As noted, which version of each statement a participant saw was also randomized (so that each participant saw and responded to half of the full set of 60 statements).

### 3.2. Results and Discussion

**Figure 4:**
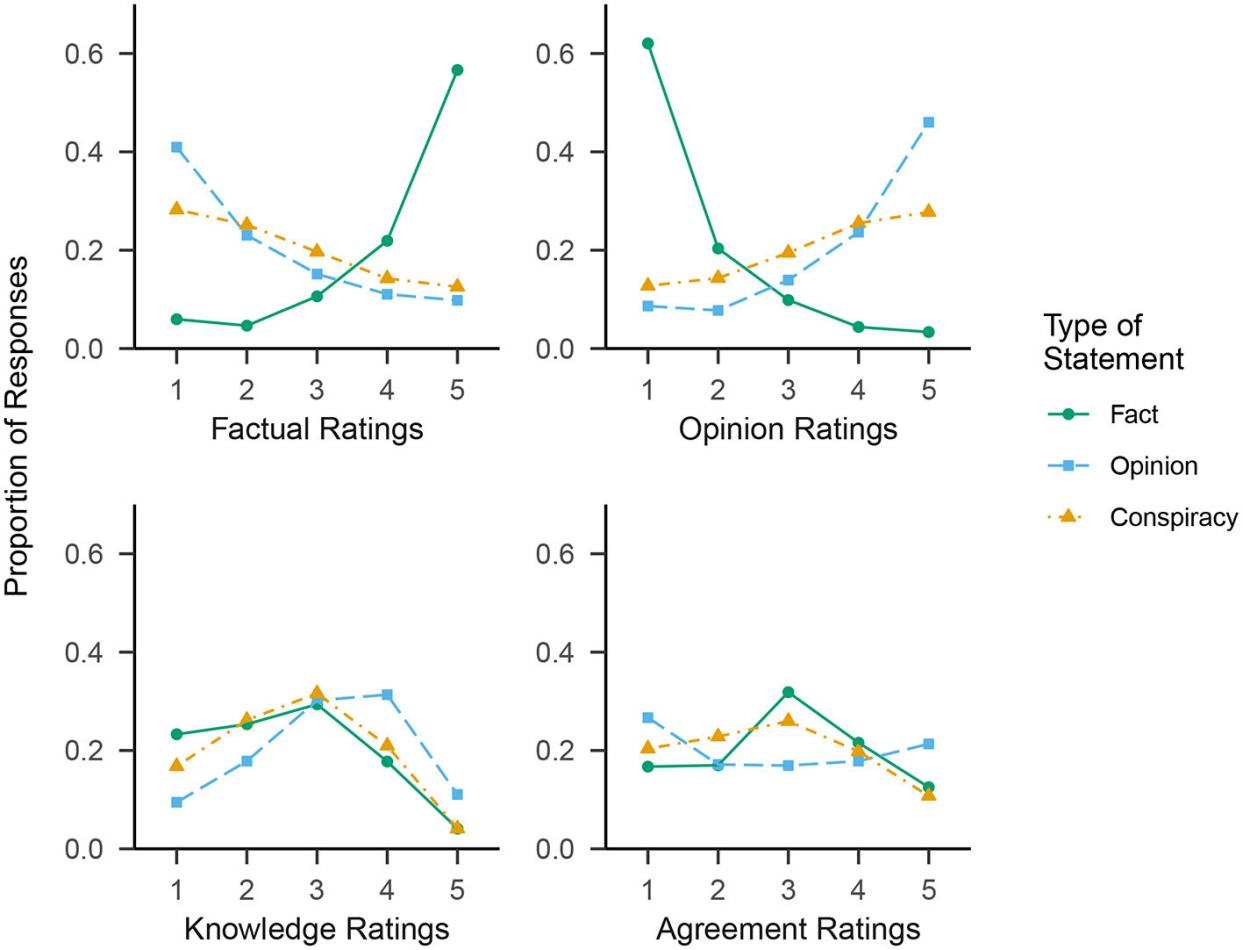
Proportions of ratings at each point on the 5-point response scale to each of the Factual, Opinion, Knowledge, and Agreement rating scales in Study 2. The three separate lines in each panel represent the three different types of stimulus statement: Fact, Opinion, and Conspiracy.

First, we produced plots showing the separate distributions of responses for each of the three statement types on each of the four rating scales (Figure 4). As each participant provided 10 ratings per statement type for each rating scale, the maximum number of data points for each distribution is N*10 = 1, 460 (< 1% of data was missing per distribution; the actual range was 1,454–1,460).

*Factual* and *opinion* ratings were strongly skewed. For statements of fact, *factual* ratings were negatively skewed; the highest two response options account for the majority of responses. *Opinion* ratings skew in the opposite direction. Conversely, for statements of opinion, *factual* ratings were positively skewed and *opinion* ratings negatively skewed, though the skew is less pronounced than for statements of fact. In short, this suggests that participants more often than not accurately labeled statements of fact as factual and statements of opinion as opinion-based.

Statements of conspiracy exhibit skew in the same direction as statements of opinion, though the skew is substantially less pronounced. That is, in terms of the sheer number of ratings across the *factual* and *opinion* scales, conspiracy theories appear to occupy a middle ground between statements of fact and statements of opinion.

For the *knowledge* and *agreement* ratings scales, no strong skew is evident, and differences between the three statements types are less pronounced. The most noteworthy trend was for participants to claim most knowledge about statements of opinion; slightly less in regards to statements of conspiracy, and the least knowledge of statements of fact. This may be because our statements of fact generally made more specific claims than the statements of opinion, frequently involving statistics likely to be unfamiliar to most. As far as *agreement*, participants agreed most strongly with the statements of opinion overall, least strongly with the statements of conspiracy, with statements of fact in between.

Next, bivariate correlations were computed to establish the strength of association between pairs of ratings. Pearson's *r* correlation coefficients were calculated for all six possible pairwise combinations of the four rating scales, using every complete pair of ratings for each statement type (see Table 1).

**Figure 5:**
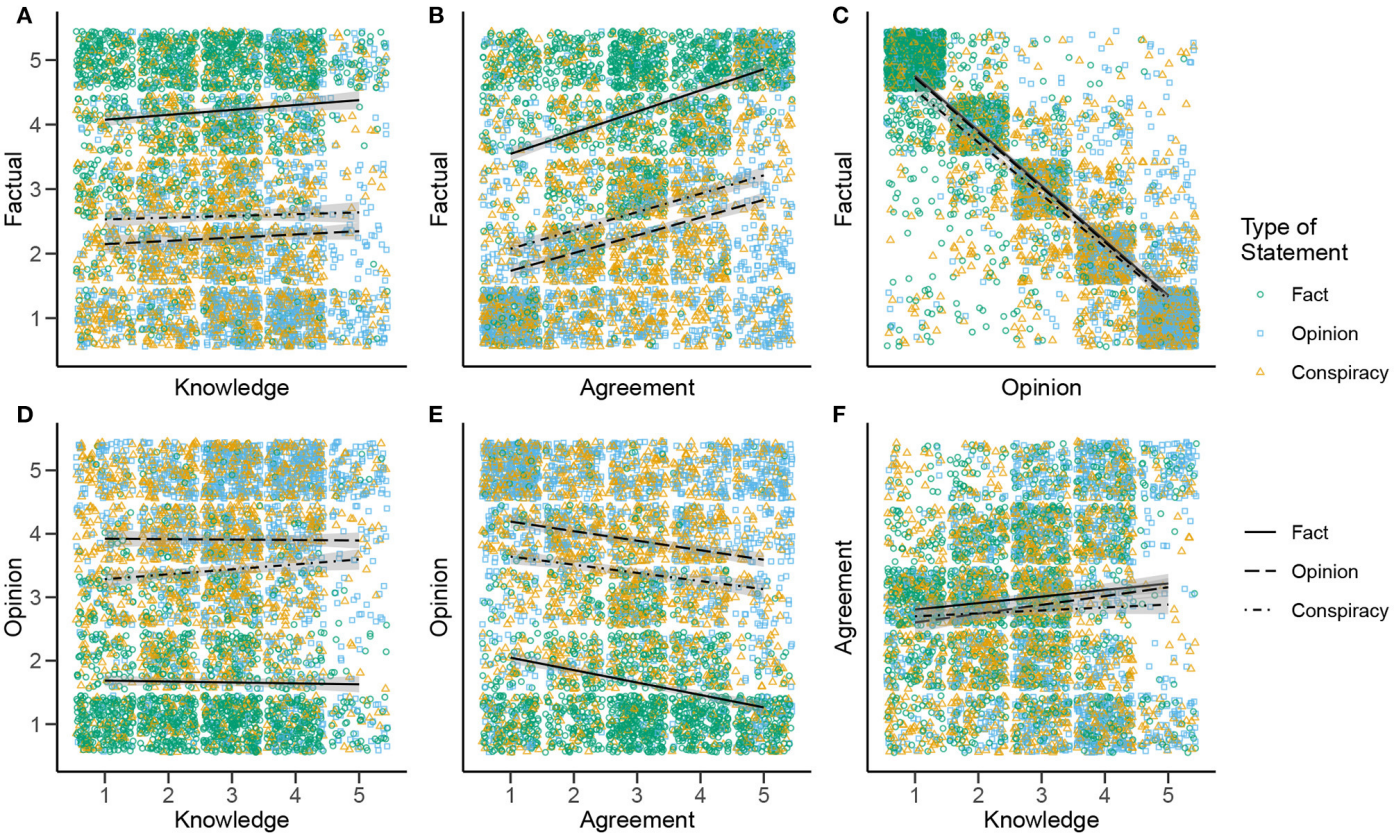
Scatterplots of bivariate relationships between Factual × Knowledge **(A)**, Factual × Agreement **(B)**, Factual × Opinion **(C)**, Opinion × Knowledge **(D)**, Opinion × Agreement **(E)**, and Agreement × Knowledge **(F)** rating scales in Study 2. The three different types of stimulus statement (Fact, Opinion, and Conspiracy) are represented as differently colored and shaped points. Linear trend lines are included, with different line types representing trends for the three types of stimulus statement.

To visualize these pairwise associations, we generated scatterplots for each of the six pairwise comparisons (Figure 5). In this study, knowledge appeared to play little role in labeling a statement as factual or opinion (Figures 5A,D). The most noteworthy trend is that greater self-rated knowledge weakly predicted stronger agreement with the claim—though visual inspection of the scatterplot (Figure 5F) suggests that knowledge may in fact polarize agreement ratings; participants who selected the highest rating for knowledge tended to indicate either strong agreement *or* strong disagreement, while largely neglecting the intermediate scale points.

Agreement does appear to play a role in labeling statements as factual or opinion-based (Figures 5B,E). There was a small-to-moderate positive correlation between agreement and *factual* ratings, and a weaker negative correlation for *opinion* ratings. That is, subjectively perceiving a claim to be true predicted rating it more strongly as factual and less strongly as opinion. The different magnitude of these relationships suggests, as in Study 1, an asymmetry between perceptions of factuality and opinion.

The strongest correlations were between *factual* and *opinion* ratings. Correlations for the various statements types ranged from *r* = −0.76 to *r* = −0.82. Thus, to an even greater extent than in Study 1, the labels appear to be used as mutually exclusive. This is illustrated visually in the strong clustering of points along the diagonal of the scatterplot (Figure 5C).

Perhaps most noteworthy, comparing the respective correlations across each type of statement shows no major differences (visually represented in the largely parallel slopes on each scatterplot). In other words, the trends do not differ depending on the type of statement in question. The main difference between statement types appears to be in the intercept of the lines, or the average values of the ratings. Again, the most consistent trend is that statements of fact are rated as more factual, on average, while statements of opinion were rated as more opinion, suggesting that participants were overall able to accurately label the statements. (This is evident visually in the scatterplots, where green *statements of fact* dots cluster toward higher ratings on the *factual* rating scales, while blue *statements of opinion* dots cluster toward lower ratings, and vice versa on the *opinion* rating scales.) Statements of conspiracy (orange dots in the scatterplots) consistently appear to occupy a middle ground between statements of fact and opinion, though generally leaning more toward opinion. Thus, whether one agrees or disagrees with a conspiracy theory, it is seen neither as a purely factual claim nor as mere opinion, but as something between the two.

In sum, this study suggests that, in the context of political claims of fact, opinion, and conspiracy, the more an individual perceives a claim to be factual, the less they see it as an opinion, and vice versa. This metacognitive labeling process appears to be related to how much one personally agrees with the claim. While people are generally able to correctly distinguish factual and opinion statements, a claim is more likely to be regarded as factual to the extent one is favorably disposed toward it, whereas a claim one disagrees with is more likely to be labeled an opinion. As compared with Study 1, the magnitude of the correlation is attenuated. That the relationship persists despite the efforts to clarify the orthogonality of *truth* and *factualness* in our instructions and rating scales, however, suggests that the relationship cannot be entirely accounted for by participants interpreting *factual* in the colloquial sense of *true*. Rather, it may reflect a metacognitive bias whereby the perceived factuality of a claim is a product of one's subjective agreement with its content. As in the previous study, however, there are important limitations of the stimuli and procedure which call the generalizability of the results into question.

## 4. General Discussion

This study builds on the growing body of research examining the motivated acceptance or rejection of controversial claims (Hastorf and Cantril, 1954; Kahan et al., 2012; Dunning, 2015; Ditto et al., 2019) by examining the metacognitive processes behind labeling claims as factual or opinion-based. In two studies, using both political and non-political stimulus statements, the extent to which a statement was perceived to be factual or an opinion was related to the degree to which one personally agreed or disagreed with the claim. This suggests that rather than dismissing uncongenial facts as merely mistaken, people may construe them as fundamentally less factual and more a matter of opinion, while congenial opinions may take on the luster of fact (see Mitchell et al., 2018; Washburn and Skitka, 2018; Harper and Baguley, 2019; van der Linden et al., 2020).

Beyond routine claims of fact and opinion, this study also examined how claims of conspiracy are rated within the fact/opinion paradigm. Claims of conspiracy were consistently situated between claims of fact and opinion. This placement appears similar to previous findings that religious claims occupy a middle-ground between fact and opinion in terms of how biologically based, personally-revealing, and open to personal differences such claims are (Heiphetz et al., 2013, 2014, 2017). This also aligns with research suggesting that conspiracy thinking is driven in a top-down way by a mindset which posits that any “official stories” are not to be trusted (e.g., Wood et al., 2012). In this sense, it is possible that an individual's engagement with conspiracy theories, and perhaps “fake news” and political misinformation more broadly, is more akin to an article of ideological faith than a claim of factual knowledge or personal opinion.

Whether individuals would agree that their stance on such matters is ideological is another question. In professing to deny knowledge in order to make room for faith, Kant (1781) suggested that some domains of belief, such as religious faith—are unamenable to the same epistemological standards as objective knowledge or personal opinion. However, this perhaps reflects an idealized epistemology in which clear distinctions between fact, opinion, and faith can be drawn. In everyday reasoning, the boundaries may be more malleable. While endorsement of conspiracy theories, allegations of fake news, and other contested claims may be to some extent ideological, it may not appear so to the percipient. In the current data, labeling conspiracy theories as factual or opinion was associated with the percipient's subjective agreement with the claim to the same extent as for statements of pure fact and opinion. However, the possibility of explicitly labeling claims as ideological is not directly addressed by the current data. Future research might productively expand on this by having participants rate the extent to which a statement reflects an article of faith/ideology in addition to rating it as factual or opinion.

Future research might also explore in greater depth the role of ability and motivation in classifying claims as fact or opinion. Our research simply shows how people might categorize facts and opinions; it does not address the question of whether people care to be correct. Previous research suggests that individuals differ in accuracy motives (Pennycook et al., 2014, 2020). That is, some people may be more dispositionally inclined to seek to hold accurate beliefs rather than seeking the validation of reinforcing existing beliefs. Perhaps this extends to seeking to correctly classify claims as factual or opinion. Other research suggests that susceptibility to partisan fake news may be better explained by lack of reasoning than by motivated reasoning (Pennycook and Rand, 2019). Classifying the epistemic nature of complex statements is undoubtedly cognitively taxing. In situations of ambiguity, people may simply decline to give the issue much thought. Exploring such questions would add much to the research reported here, particularly given that it used convenience samples of undergraduates. College students may have different epistemologies and related motivations than the average member of the mass public, and are regularly evaluated on the quality and accuracy of their beliefs. How transferable such qualities are to the judgments of mundane and political statements presented in the current studies remains to be seen, as does the extent to which more representative samples of the public would result merely in different intercepts rather than a different model entirely.

In sum, the current research suggests that, while people make accurate distinctions between statements of fact and of opinion on the whole, the process by which we categorize a claim is influenced by our subjective agreement with the claim. If the findings reported here are generalizable, there are potential implications for understanding seemingly intractable political debates, particularly when it comes to hotly contested claims, such as conspiracy theories. Such debates may represent not just disagreement over the facts, but different perceptions of whether any particular claim or counter-claim is fundamentally factual. When a factual claim is disagreeable it may be seen not merely as wrong, but as biased conjecture. On the other hand, when an opinion is congenial, it may be seen not as an opinion open to differing points of view but as a matter of objective fact not up for debate. Understanding contentious claims, such as conspiracy theories and accusations of “fake news” on these terms may help understand why and for whom such claims are more or less evidentially vulnerable (*cf*. Van Leeuwen, 2014; Levy, 2017). The limited influence of “fact-checking” efforts (Nyhan et al., 2019) may be due, in part, to differing perceptions of what information is factual and what is opinion.

Yet it must be reiterated that the studies reported here are exploratory and descriptive by design, rather than setting out to confirm specific hypotheses. The findings should thus be considered preliminary and subject to further examination. Limitations of the *ad-hoc* stimuli and the politically and demographically homogeneous participant samples, in particular, prohibit strong claims of generalizability. More systematic research is required to further map the contours of how we think about facts, opinions, and conspiracy theories. The current tentative findings suggest that exploring metacognition may be a productive avenue for further research.

## Data Availability Statement

The raw data supporting the conclusions of this article will be made available by the authors, without undue reservation.

## Ethics Statement

The studies involving human participants were reviewed and approved by Institutional Review Boards of Barnard College and Ajou University. The patients/participants provided their written informed consent to participate in this study.

## Author Contributions

RB and LS contributed equally to the conception and design of the work, the acquisition, analysis, and interpretation of data, and to drafting and revising this manuscript.

## Conflict of Interest

The authors declare that the research was conducted in the absence of any commercial or financial relationships that could be construed as a potential conflict of interest.

## Footnotes

^1^All stimuli and response scales were also translated into Korean by bilingual English and Korean speakers, and a separate sample of 215 participants was recruited in South Korea. The trends were largely consistent with those reported here, with some differences in item descriptives and correlation magnitudes. It is unclear whether such differences reflect meaningful psychological differences or idiosyncrasies of how the stimuli and instructions function across the two languages. As it neither adds nor detracts from the conclusions that can be drawn, analysis of this data is presented as Supplementary Material.
