# Supplementary material for: Metacognitive Labeling of Contentious Claims: Facts, Opinions, and Conspiracy Theories
Source: Front Psychol. 2021 Mar 25;12:644657. doi: 10.3389/fpsyg.2021.644657 (PMC8044776; doi:10.3389/fpsyg.2021.644657)
Supplement: Supplementary file 1 [file Data_Sheet_1.PDF]

## Supplementary Material

## 1 SUPPLEMENTARY DATA

The 50 stimulus statements used in Study 1 were translated into Korean by fluent bilingual speakers, and a separate sample of 202 Korean-speaking students (72.77% female) was recruited at Ajou University in South Korea (13 additional participants did not complete the procedure). These participants completed the same procedure detailed in Study 1.

The general trends were largely consistent with those reported for the USA sample in the main text, with some differences in item descriptives and correlation magnitudes. For example, looking at the average Fact, Agreement, and Opinion ratings for each of the 50 statements (see Figure S1) shows that the Fact and Agreement ratings are more strongly coupled than among the USA sample, with the black Fact ratings and red Agreement ratings corresponding closely. This is reflected in the stronger correlation between the two ratings (see Table S1). On the other hand, the grey Opinion ratings are a less symmetrical mirror image of the black Fact ratings than they were among the USA sample, corresponding to a weaker negative correlation.

Looking at the histograms of all responses to all 4 response scales (Figure S2) shows that responses were more evenly distributed across the scale for the sample from South Korea, particularly for Fact and Opinion ratings when compared to the heavily skewed distributions for the USA sample. The more tempered use of the scales is evident in the 2-dimensional histograms of ratings for each pair of scales (Figure S3).

It is unclear whether such differences reflect metacognitive differences in categorizing statements as fact and opinion, or differences in language use; the current exploratory, descriptive findings are not equipped to establish the cause of the minor discrepancies. Yet the overall picture is that the general trends among the Korean speaking students in Korea are largely similar to those from the sample of English speakers obtained at a USA college detailed in Study 1, in line with the general argument that claims are more likely to be labeled factual to the extent that one subjectively agrees with the claim.

## 2 TABLE

**Table S1.** Pearson's correlation coefficients for USA and South Korea (KO) samples.

| Pairwise Comparison | USA | KO |
|---|---|---|
| Factual x Opinion | -0.52 | -0.26 |
| Factual x Knowledge | 0.22 | 0.28 |
| Factual x Agreement | 0.52 | 0.74 |
| Opinion x Knowledge | -0.13 | -0.08 |
| Opinion x Agreement | -0.38 | -0.22 |
| Knowledge x Agreement | 0.22 | 0.28 |

## 3 FIGURES

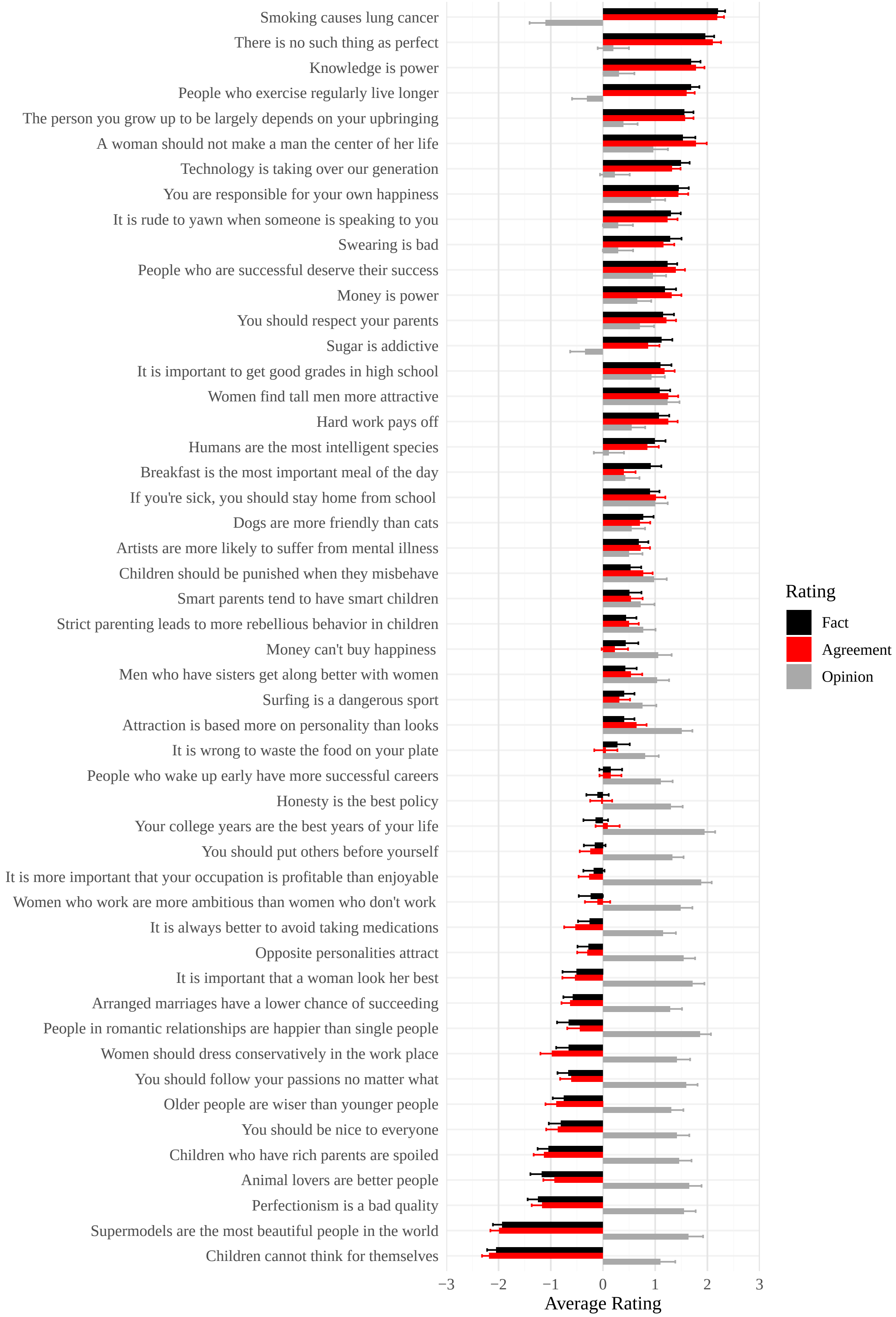


**Figure S1.** Average fact, opinion, and agreement ratings for each of the fifty statements.

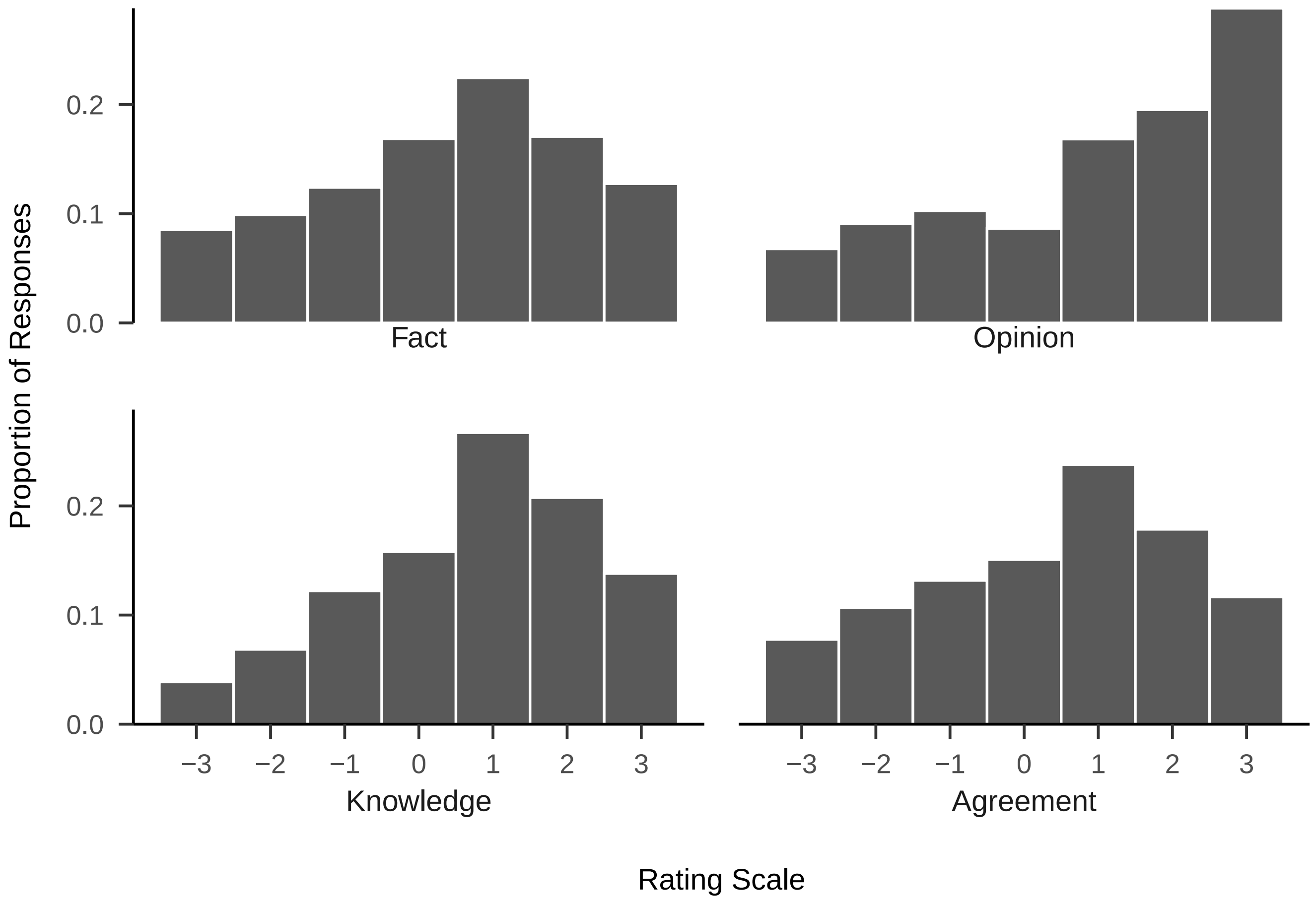


**Figure S2.** Histograms of all responses across rating scales.

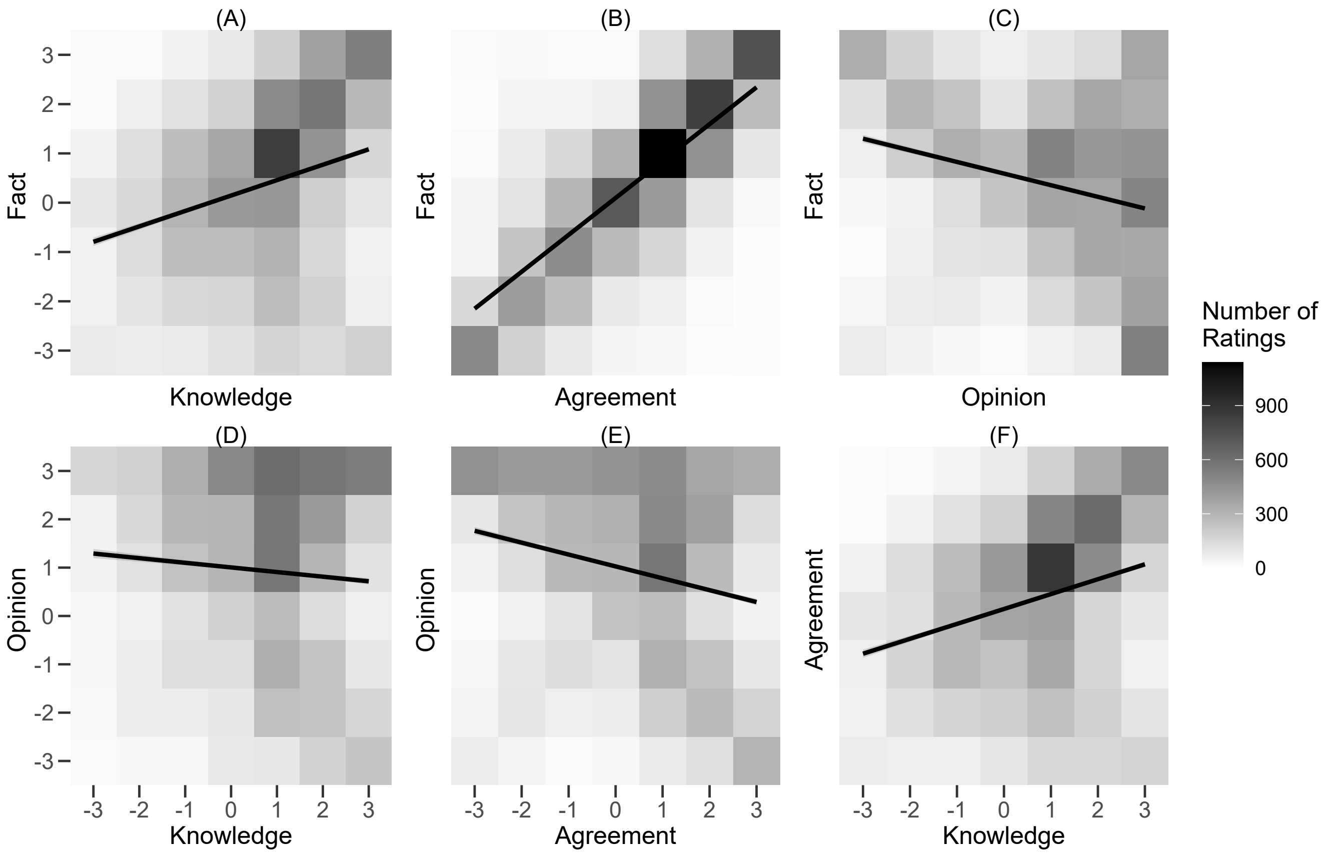


**Figure S3.** 2-dimensional histograms of pairwise associations between rating scales
