# Supplementary material for: Metacognitive Labeling of Contentious Claims: Facts, Opinions, and Conspiracy Theories
Source: Front Psychol. 2021 Mar 25;12:644657. doi: 10.3389/fpsyg.2021.644657 (PMC8044776; doi:10.3389/fpsyg.2021.644657)
Supplement: Supplementary file 2 [file Data_Sheet_2.pdf]

Appendix

Complete wording of stimuli used in Study 2.

Topic	Category	Statement Version A	Statement Version B
Pharma	Fact	The pharmaceutical industry has the largest lobby in congress.	The pharmaceutical industry does not have the largest lobby in congress.
	Opinion	The pharmaceutical industry is driven only by profit.	The pharmaceutical industry is driven only by the desire to help citizens.
	Conspiracy	The pharmaceutical industry purposefully over-prescribes drugs to increase addictions and profit.	The pharmaceutical industry does not purposefully over-prescribe drugs to increase addictions and profit.
Climate	Fact	Global temperatures have risen more than 2 degrees Fahrenheit since 1900.	Global temperatures have risen less than 2 degrees Fahrenheit since 1900.
	Opinion	Climate change is an existential threat.	Climate change is not an existential threat.
	Conspiracy	The scientific consensus about climate change is distorted by scientists' own interests	The scientific consensus about climate change is not distorted by scientists' own interests
Economy	Fact	The United States GDP has risen 5% since 2016.	The United States GDP has fallen 5% since 2016.
	Opinion	Trickle-down economics harms working-class Americans and the national economy	Trickle-down economics benefits working-class Americans and the national economy.
	Conspiracy	The economy is controlled by a small, secret group of people.	The economy is not controlled by a small, secret group of people.
Immigration	Fact	Undocumented/illegal immigrants commit crimes at a higher rate than most Americans.	Undocumented/illegal immigrants commit crimes at a lower rate than most Americans.
	Opinion	Undocumented immigrants have valid reasons for entering the US illegally.	Undocumented immigrants do not have valid reasons for entering the US illegally.

	Conspiracy	The government downplays the true cost of immigration to taxpayers and society.	The government exaggerates the true cost of immigration to taxpayers and society.
Russia	Fact	Russian intelligence agents hacked American voting machines in the 2016 election.	Russian intelligence agents did not hack American voting machines in the 2016 election.
	Opinion	Donald Trump's diplomatic relationship with Russia poses a threat to our nation.	Donald Trump's diplomatic relationship with Russia does not pose a threat to our nation.
	Conspiracy	Donald Trump is being used as a puppet for Vladimir Putin.	Donald Trump is not being used as a puppet for Vladimir Putin.
Police	Fact	More black people than white people were killed by police in 2018.	More white people than black people were killed by police in 2018.
	Opinion	The criminal justice system is fundamentally unfair.	The criminal justice system is fundamentally fair.
	Conspiracy	Issues of police brutality are deliberately sensationalized to further racial divides.	Issues of police brutality are not deliberately sensationalized to further racial divides.
Gun control	Fact	The number of gun homicides is currently at an all-time high.	The number of gun homicides is currently lower than it has been in the past.
	Opinion	Access to guns should be more restricted.	Access to guns should be less restricted.
	Conspiracy	Some mass shootings have been staged by the government.	No mass shootings have been staged by the government.
Abortion	Fact	The number of abortions performed in the US annually is at an all-time high.	The number of abortions performed in the US annually is at an all-time low.
	Opinion	Abortion is equivalent to taking an innocent life.	Abortion is not equivalent to taking an innocent life.
	Conspiracy	Abortion is deliberately encouraged to reduce certain populations.	Abortion is not deliberately encouraged to reduce certain populations.
Terrorism	Fact	In the US, there have been more terrorist attacks committed in the past year by white men than by Middle Eastern men.	In the US, there have been more terrorist attacks committed in the past year by Middle Eastern men than by white men.

	Opinion	Domestic terrorists pose a greater threat to the US than terrorists from the Middle East.	Terrorists from the Middle East pose a greater threat to the US than domestic terrorists.
	Conspiracy	The media deliberately sensationalizes terror attacks by foreign attackers as compared to domestic terrorists.	The media deliberately sensationalizes terror attacks by domestic terrorists as compared to foreign attackers.
War	Fact	Following the US invasion of Iraq in 2003, US forces did not find weapons of mass destruction.	Following the US invasion of Iraq in 2003, US forces found weapons of mass destruction.
	Opinion	United States military intervention in the Middle East is a justifiable foreign policy	United States military intervention in the Middle East is not a justifiable foreign policy
	Conspiracy	The government has not always been open about its real reasons for declaring war in the Middle East.	The government has always been open about its real reasons for declaring war in the Middle East.
